# Eosinophilic Vacuolated Tumor of Kidney: Clinical Perspective of a New Pathologic Emerging Entity

**DOI:** 10.7759/cureus.24716

**Published:** 2022-05-04

**Authors:** İbrahim Kartal, Halil Ibrahim Ivelik, Şeref Çoşer, Hazal Tunç, Mustafa Fuat Açıkalın

**Affiliations:** 1 Urology, Kütahya Health Science University, Kütahya, TUR; 2 Urology, Kutahya Health Science University, Kütahya, TUR; 3 Pathology, Eskisehir Osmangazi University, Eskişehir, TUR; 4 Pathology and Laboratory Medicine, Eskisehir Osmangazi University, Eskişehir, TUR

**Keywords:** oncocytoma, nephron-sparing treatment, kidney, hybrid malignancy, vacuolated tumor, eosinophilic

## Abstract

The eosinophilic vacuolated tumor (EVT) of the kidney is a newly identified and pathological emerging entity. In this case report, EVT diagnosed due to a partial nephrectomy performed for a suspicious kidney mass in a 47-year-old patient is presented. A review of the literature and this case indicates that EVT, also called high-grade oncocytoma, does not show clinically aggressive behavior. However, in case of clinical suspicion, tumors with this oncocytic morphology should be treated with nephron-sparing treatment methods, considering that they may be hybrid malignancies.

## Introduction

Renal masses are common pathologies in the urology clinic, which can be diagnosed incidentally with the frequent use of radiological imaging methods. A pathological diagnosis is crucial for evaluating the follow-up and treatment processes of patients from a urological point of view. Therefore renal masses that can not be classified can cause clinical difficulties. Sometimes, unnecessary invasive treatments applied to these masses may cause severe morbidities [1].

The 2016 World Health Organization (WHO)/International Society of Urological Pathology (ISUP) classification is used to classify renal masses, in which more than 50 pathologies are defined. Included under the title of the novel, emerging and provisional renal entities are recently defined in this case report and are not yet defined in the WHO/ISUP classification. in our case report, the preoperative examination and patient characteristics of our patient, who was diagnosed with eosinophilic vacuolated tumor (EVT) [2] pathologically after partial nephrectomy, were reviewed. As far as we know, the emerging entity EVT has not been evaluated urologically in the literature until now. This case report has been prepared to examine the preoperative clues of the EVT, present its perioperative features, and evaluate its postoperative prognosis.

## Case presentation

**Figure 1:**
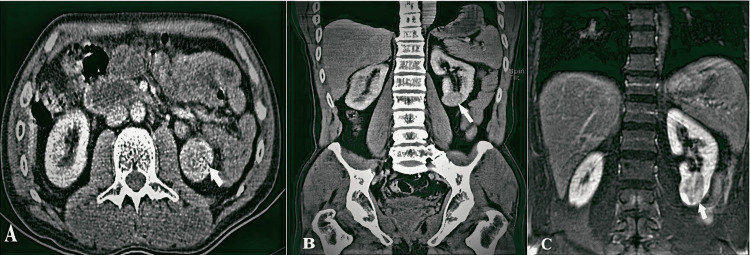
Eeosinophilic vacuolated tumor in a 47-year-old man A-B: Computed tomography transverse and coronal sections showing well-defined round heterogenous enhancement of the left lower pole renal mass (white arrows); C. Coronal contrast-enhanced T1-weighted image shows heterogeneous enhancement in the left lower pole renal mass (white arrow).

A 47-year-old male patient with no known comorbidity presented with left flank pain. The patient's physical examination was considered normal, and his history included being followed up for urolithiasis in the left kidney seven years ago and spontaneous stone passage. He is a teacher with no history of risky occupational exposure. The patient, who was admitted to the physical therapy and rehabilitation clinic due to flank and back pain in the last year, was found to be normal in a complete urinalysis, while Hgb: 16.1 g/dL, creatinine: 0.9 mg/dL. During the urinary ultrasonography at the beginning of the urological evaluation, an isoechoic solid lesion measuring 40x30 mm with a lobulated contour including internal vascularity was observed in the lower pole of the patient's left kidney. Contrast-enhanced abdomen computed tomography (CT) of the patient, who was initially evaluated as renal cell carcinoma (RCC), revealed a solid character of 33x27 mm in the left kidney lower pole medial aspect with medium contrast, and a well-contoured soft tissue mass suggesting a tumoral mass (Figure 1A-1B). In addition, magnetic resonance imaging of the upper abdomen showed a 38 mm diameter, nonhomogeneous, relatively smooth-contoured nodular mass in the lower pole of the left kidney. At the same time, contrast-enhanced examination revealed low-intensity nonhomogeneous enhancement in the mass (Figure 1C). It was reported as RCC. The nephrometry scores showed that the mass was of low complexity. Possible treatment modalities were explained to the patient in light of the current findings. A partial nephrectomy was planned for the renal mass.

In the operation, a soft, well-circumscribed but non-encapsulated mass of approximately 4 cm was seen in the lower pole of the kidney. While it was observed that the separation of the mass from the surrounding tissue was not clear during the resection, the resection was performed so that the surgical margin was negative. The surgery was completed with a warm ischemia time of 11 minutes and 100 cc of bleeding in a total of 80 minutes. The patient, whose general condition was good after the operation and was followed up clinically, was discharged on the second postoperative day.

**Figure 2:**
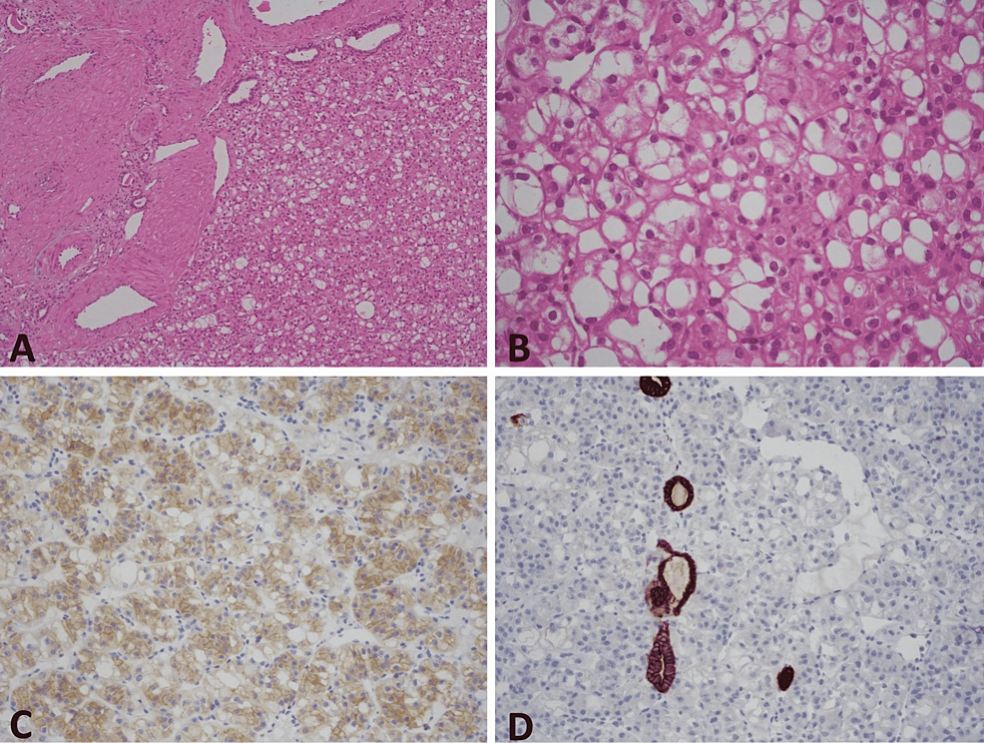
A. Tumor shows a solid growth pattern with large thick-walled vessels and entrapped non-neoplastic tubules at the periphery (hematoxylin and eosin x100). B. Tumor cells exhibit voluminous eosinophilic cytoplasm with prominent intracytoplasmic vacuoles. The cells have round nuclei and prominent nucleoli (hematoxylin and eosinx200). C. Tumor cells are positive for CD117 (x100). D. CK7 is negative in tumor cells. Only entrapped non-neoplastic tubules are positive for CK7 (x100).

Macroscopically, the tumor was well-circumscribed, smooth, and mahogany brown. Microscopically, the tumor consisted of cells with a solid or occasionally nesting pattern, with extensive eosinophilic cytoplasm (Figure 2A). There were prominent intracytoplasmic vacuoles in tumoral cells (Figure 2B). Tumor cell nuclei were round or oval and contained prominent eosinophilic nucleoli. Thick-walled vascular structures and non-neoplastic tubules trapped in the tumor were observed in the peripheral parts of the tumor. In the immunohistochemical evaluation, PAX 8, CD117 (KIT) (Figure 2C), and CD10 were diffuse and alpha-methylacyl-CoA racemase focal positive in tumoral cells. Succinate dehydrogenase-B expression was preserved. CK7 (Figure 2D), carbonic anhydrase IX, vimentin, RCC marker, human melanoma black 45, and MART-1 were negative. With the histopathological and immunohistochemical findings described, the mass was reported as a recently defined EVT, which was not included in the 2016 WHO classification. It was decided to apply a follow-up protocol suitable for RCC low-risk recurrence, to follow up on this new pathological variant, which does not have long-term patient data in terms of urology. Contrast-enhanced abdominal CT performed at the six-month follow-up showed no recognizable mass in the ipsilateral left kidney, and the patient's glomerular filtration rate was within normal values.

## Discussion

There are insufficient data available to predict the clinical behavior of this newly identified EVT. During the evaluation of our case and the literature, it was seen that EVT exhibits clinically nonaggressive behaviors. The anatomical and radiological features of the reported masses are also consistent with the clinical non-aggressiveness of EVT. However, in case of clinical suspicion or detection in renal biopsy, it would be appropriate to treat EVT with nephron-sparing methods until high-level evidence is obtained.

According to the case series by He et al. and Chen et al., it was found that EVT is more common in women, unlike in our case. All the cases in these studies were operated on at the clinical T1 stage. Moreover, recently, Farcaş et al. reported epidemiological findings similar to the first two studies in their study. Although the mean follow-up period of these studies was not very long, no recurrence or death was observed in the follow-up period of up to 198 months in a total of 40 patients in these three studies [3-5]. Although Chen et al. first defined EVT as "sporadic renal cell carcinoma with eosinophilic and vacuolated cytoplasm", in a recent case report, a 48-year-old patient with tuberous sclerosis was also identified in a syndromic case. However, he did not display aggressive behavior [6]. It has been seen that EVT is also defined as an oncocytic tumor in some cases.

Radiologic and anatomical predictors can distinguish benign from malignant such as nephrometry scores and vena cava lesion-attenuation difference; the lesion-cortex-attenuation ratio was evaluated retrospectively for our case, and no clue could be obtained. Renal mass biopsy (RMB) may be recommended for active surveillance in cases where EVT is suspected. However, the presence of renal oncocytoma in the RMB does not exclude malignancy. It should be kept in mind that there may be hybrid tumors with oncocytomas [7]. In case of suspicion, it would be reasonable to treat EVTs that do not currently have evidence of aggressive behavior and are predicted to have low radiologically low nephrometric scores with nephron-sparing approaches as much as possible.

The use of 99Tc-sestamibi single-photon emission computed tomography (SPECT/CT) is promising, in line with the studies performed on the differentiation of renal oncocytoma and RCC [8]. It could be predicted that 99Tc-sestamibi uptake would be high in our case. However, 99m Tc-sestamibi SPECT/CT was not performed on our patient since surgery was performed with a preliminary diagnosis of RCC.

Another finding that can be used in the clinical approach to EVT is the association of EVT with mTOR pathway abnormalities. In this context, nonoverlapping mutations have been observed in tuberous sclerosis complex 1, tuberous sclerosis complex 2, and the mammalian target of rapamycin (mTOR) genes [5,9]. Although EVT is not defined accurately as a carcinoma, for now, the use of mTOR inhibitors will probably be considered as the first line of treatment when systemic treatment for EVT is required.

## Conclusions

EVT is a newly defined entity with insufficient information, which urologists should be aware of. In our case report and the cases reported so far, EVT did not exhibit aggressive behavior. EVT should be treated with nephron-sparing methods until a high level of evidence for the treatment scheme is available, along with regular follow-up. A multidisciplinary approach, including the pathologists, should be applied.

## References

[1] Abdessater M, Kanbar A, Comperat E, Dupont-Athenor A, Alechinsky L, Mouton M, Sebe P (2020). Renal oncocytoma: an algorithm for diagnosis and management. Urology.

[2] Trpkov K, Williamson SR, Gill AJ (2021). Novel, emerging and provisional renal entities: The Genitourinary Pathology Society (GUPS) update on renal neoplasia. Mod Pathol.

[3] He H, Trpkov K, Martinek P (2018). "High-grade oncocytic renal tumor": morphologic, immunohistochemical, and molecular genetic study of 14 cases. Virchows Arch.

[4] Chen YB, Mirsadraei L, Jayakumaran G (2019). Somatic mutations of TSC2 or MTOR characterize a morphologically distinct subset of sporadic renal cell carcinoma with eosinophilic and vacuolated cytoplasm. Am J Surg Pathol.

[5] Farcaş M, Gatalica Z, Trpkov K (2022). Eosinophilic vacuolated tumor (EVT) of kidney demonstrates sporadic TSC/MTOR mutations: next-generation sequencing multi-institutional study of 19 cases. Mod Pathol.

[6] Trpkov K, Bonert M, Gao Y (2019). High-grade oncocytic tumour (HOT) of kidney in a patient with tuberous sclerosis complex. Histopathology.

[7] Deledalle FX, Ambrosetti D, Durand M (2021). Active surveillance for biopsy proven renal oncocytomas: outcomes and feasibility. Urology.

[8] Almassi N, Gorin MA, Purysko AS (2018). Use of 99mTc-sestamibi single-photon emission computed tomography / X-ray computed tomography in the diagnosis of hybrid oncocytic / chromophobe tumor in a pediatric patient. Urology.

[9] Siadat F, Trpkov K (2020). ESC, ALK, HOT and LOT: three letter acronyms of emerging renal entities knocking on the door of the WHO classification. Cancers (Basel).

